# Effect of Herbal Formulation on Immune Response Enhancement in RAW 264.7 Macrophages

**DOI:** 10.3390/biom10030424

**Published:** 2020-03-09

**Authors:** Tuy An Trinh, Jimin Park, Ji Hong Oh, Jung Sik Park, Dahae Lee, Chang Eop Kim, Han-Seok Choi, Sang-Back Kim, Gwi Seo Hwang, Bon Am Koo, Ki Sung Kang

**Affiliations:** 1 College of Korean Medicine, Gachon University, Seongnam 13120, Korea; tuyantrinh@gmail.com (T.A.T.); jihong421@hanmail.net (J.H.O.); lucidpjs@naver.com (J.S.P.); pjsldh@naver.com (D.L.); eopchang@gachon.ac.kr (C.E.K.); seoul@gachon.ac.kr (G.S.H.); 2 New Drug Research Team, Kolmar Korea Co. Ltd, Sejong-si 30003, Korea; jimpark@kolmar.co.kr (J.P.); fcosmos@kolmar.co.kr (H.-S.C.); m302@kolmar.co.kr (S.-B.K.)

**Keywords:** *Saussurea lappa*, *Terminalia chebula*, *Zingiber officinale*, nitric oxide, immune response enhancement, anti-cancer

## Abstract

Immune response is a necessary self-defense mechanism that protects the host from infectious organisms. Many medicinal plants are popularly used in Asian folk medicine to increase body resistance. An herbal formulation named KM1608 was prepared from three medicinal plants: *Saussurea lappa*, *Terminalia chebula*, and *Zingiber officinale*. In this study, we evaluated the immune stimulatory effect of KM1608 on RAW 264.7 murine macrophages. Network pharmacological analyses were used to predict potential immune response pathways of major compounds from KM1608. The cytotoxicity and immuno-stimulating effect of KM1608 were determined using cell viability and nitric oxide assays. The underlying mechanism of immunomodulatory activity was evaluated by quantitative real-time reverse transcription polymerase chain reaction (qRT-PCR) of pro-inflammatory cytokines. The results of network pharmacological analysis suggested that major compounds from KM1608 possess anticancer potential via immune signaling pathways. After treatment with KM1608 at 25–100 µg/mL for 24 h, the level of nitric oxide was increased in the dose-dependent manner. The results of quantitative real-time PCR showed that KM1608 stimulates the expression of immune cytokines (interferon (IFN)-α, -β, IL-1β, -6, IL-10, inducible nitric oxide synthase (iNOS), and cyclooxygenase-2 (COX-2)) in macrophages. KM1608 extract is a potential agent for immune response enhancement.

## 1. Introduction

Immune response is an important self-defense mechanism that protects the host from numerous pathogenic infections. An immune response includes innate and adaptive immunity. Innate immune response occurs immediately when the infectious agents approach the external barrier, whereas the adaptive response causes the formation of immunological memory that allows a quicker and more effective response upon next encounter with the same pathogen [1]. Abnormality or dysfunction of the immune system may cause an unexpected immune response in the case of hypersensitivity or autoimmunity, as well as immunodeficiency. In addition, malnutrition or immune ageing results in an ineffective immune response. Immunity may be impaired by certain pathogens, such as human immunodeficiency virus (HIV), or in the patients who suffer from immunosuppression at the advanced stage of some chronic diseases [2].

Immunotherapy has long been applied for cancer treatment. The role of the immune system in the interaction with cancer cells is most clearly reflected through anti-tumor immune responses. In the tumor microenvironment, the death cells will release damage-associated molecular patterns, which stimulates tumor-infiltrating antigen-presenting cells to produce type-I interferons (IFNs). IFNs activate CD8α+ dendritic cells to present tumor-associated antigens to CD8+ T-cells. The activated CD8+ T-cells are then recruited to the tumor site to control tumor growth. In cancer treatment, both chemo- and radio-therapy induce tumor cell death via signaling pathways relevant to the immune response [3].

In the present study, we prepared a herbal formulation named KM1608, using three medicinal plants: *Saussurea lappa* C. B. Clarke (also known as *Aucklandia lappa* DC.), *Terminalia chebula* Retz, and *Zingiber officinale* Roscoe. These herbs are popularly used in Asian folk medicine for the treatment of common diseases such as cold, fever, sore throat, cough, indigestion, vomiting, and diarrhea [4,5,6]. Previous studies have revealed anticancer [7,8,9], anti-inflammatory [10,11], anti-microbial, anti-parasitic, and anti-feedant [4] activities of *S. lappa*. *Terminalia chebula* has been reported for its antioxidant [12,13], anti-bacterial [14], anti-carcinogenic, wound healing, and immunomodulatory [5,15,16] activities. The main biological effects of *Z. officinale* were anti-nausea [17], anti-diabetic [18], anti-neoplastic [19], anti-inflammatory [20,21], anti-oxidant, and immunomodulatory [6,22]. In this study, we investigated the combined immunostimulatory effect of these medicinal plants for cancer treatment.

The aim of our study was to determine the immune response enhancement activity of KM1608 on RAW 264.7 murine macrophage cells. Network pharmacological analyses were performed to predict potential pathways of major compounds from KM1608 that are relevant to the immune response. After KM1608 treatment, the cytotoxicity and immuno-stimulating effect were determined using cell viability and nitric oxide assays. The underlying mechanism of immunomodulatory activity was clarified by evaluating the expression of cytokines, such as IFN-α, IFN-β, tumor necrosis factor-α (TNF-α), IL-1β, IL-6, IL-10, inducible nitric oxide synthase (iNOS), and cyclooxygenase-2 (COX-2).

## 2. Materials and Methods 

### 2.1. Plant Materials and KM1608 Extraction

All plant materials were purchased from Songrim Muyak (Seoul, Korea). It was authenticated by Professor Donghun Lee, Gachon University College of Korean Medicine, where the voucher specimens (D180107001) were deposited on January 2018. The KM1608 formula of *S. lappa*, *T. chebula*, and *Z. officinale* was created by mixing the respective components according to a ratio of 2:2:1 (*w/w/w*) based on the results of our previous anti-inflammatory screening on 350 samples included single extracts, mixture, and formulation of medicinal plants [23]. The mixture was refluxed twice with 50% ethanol at 80 °C for 3 h each time and filtered. The filtrate was evaporated under reduced pressure to remove solvent until 35–45% of yield was obtained. After that, it was dispersed in 50% ethanol and filtered using a 0.22 µm membrane filter to acquire 5 mg/mL KM1608 solution for high performance liquid chromatography (HPLC) analysis. In the other hand, the filtrate of KM1608 extract was evaporated and then freeze-dried to acquire the dry powder for biological assays.

### 2.2. High performance liquid chromatography-ultraviolet/ diode array detection (HPLC-UV/DAD) Conditions

Quantitative analysis of KM1608 was performed using a Waters ultra performance liquid chromatography (UPLC) system and Waters Acquity UPLC HSS T3 column (100 mm × 2.1 mm, 1.8 μm) (Waters Co., Milford, MA, USA). The mobile phase consisted of water containing 0.1% phosphoric acid (A) and acetonitrile (B). The gradient elution was as follows: 0–4 min of 45% (B), 4–5 min of 45–50% (B), 5–9.9 min of 50–100% (B) for dehydrocostus lactone and 6-gingerol, 0–6 min of 12–14% (B), 6–7 min of 14–100% (B) for ellagic acid. Following gradient elution, the column was washed with 100% (B) for 7 min. The post-running time was 10 min after restoration of the initial condition. The mobile phase flow rate was 0.7 mL/min and the injection volume was 2 μL. Analytical method of HPLC was validated as described previously [23]

### 2.3. Network Pharmacological Analyses

After verifying active phytochemicals of KM1608 by HPLC analysis, we performed an in silico network pharmacological analysis to identify the targets of compounds and their potential pathways. Target genes of the active compounds were predicted from the Traditional Chinese Medicine Systems Pharmacology (TCMSP) database [24]. We retrieved annotated pathways from the Kyoto Encyclopedia Genes and Genomes (KEGG) database [25] using the target data. Then, the pathways were listed in descending order according to their combined score, leaving only the pathways satisfying the threshold (adjusted *p*-value ≤ 0.05 and combined score ≥ 30). We visualized a network graph using Cytoscape 3.5.1 [26] by collecting information on active compounds of KM1608, their targets, and related KEGG pathways.

### 2.4. Cell Culture

The RAW 264.7 murine macrophage cells were obtained from the American Type Culture Collection (ATCC, Rockville, MD, USA). The cells were cultured in Dulbecco’s Modified Eagle medium (DMEM) medium (Cellgro, Manassas, VA, USA) supplemented with 10% fetal bovine serum (Gibco BRL, Carlsbad, MD, USA) and 1% penicillin-streptomycin (Invitrogen, Grand Island, NY, USA). The cells were maintained at 37 °C in a humidified atmosphere of 5% CO_2_ and sub-cultured every two days.

### 2.5. Cell Viability Assay

RAW 264.7 cells were seeded in a 96-well plate at the density of 1 × 10^5^ cells/well and grown for 24 h. The next day, cells were treated with 0.5% dimethyl sulfoxide (DMSO) (Sigma-Aldrich, St. Louis, MO, USA), which served as a control, or KM1608 at the concentrations of 25, 50, and 100 µg/mL. After 24 h, 10 µL of EZ-Cytox reagent (DoGen, Seoul, Korea) was added to each well, followed by 30 min incubation. EZ-Cytox reagent contains a highly water-soluble tetrazolium salt, which is reduced by dehydrogenase activities in cells to give a yellow-color formazan dye. The amount of the formazan dye, generated by the activities of dehydrogenases in cells, is directly proportional to the number of living cells. Cell viability was determined through the change of absorbance measured at 450 nm using PowerWave XS microplate reader (Bio-Tek Instruments, Winooski, VT, USA) [27].

### 2.6. Nitric Oxide Assay

Nitric oxide production was evaluated indirectly by measuring the concentration of accumulated nitrite—the final inert product of nitric oxide metabolism—in the culture medium. RAW264.7 cells (1 × 10^5^ cells/well on 96-well plate) were treated with the phenol red-free medium containing 0.5% DMSO (control) or KM1608 at the indicated concentrations for 24 h. The cell culture supernatant was collected and mixed with Griess reagent (supplemented with 1% sulfanilamide, 5% phosphoric acid, and 0.1% *N*-(1-naphthyl)-ethylenediamine) at the ratio of 1:1 (*v/v*) [28]. After incubation at room temperature for 10 min, the absorbance of the mixture was measured at 450 nm with a microplate reader. The nitrite concentration in the supernatant was calculated from a sodium nitrite standard reference curve. Prednisolone (PDS) and 5-aminosalicylic acid (5-ASA) were used as reference drugs.

### 2.7. Quantitative Real-Time Reverse Transcription polymerase chain reaction (qRT-PCR)

The RAW 264.7 cells were harvested after 12 h of treatment with 0.5% DMSO (control) or KM1608 at the indicated concentrations. Total cellular RNA was isolated with TRIzol reagent (Invitrogen, Carlsbad, CA, USA) as per the manufacturer’s instructions. The isolated RNA was reverse transcribed into complementary DNA (cDNA) using the RevertAid First Strand cDNA Synthesis Kit (Thermo Fisher Scientific, Waltham, MA, USA). The reaction mixture was prepared by mixing cDNA with sense and antisense primers in PowerUp Green Master Mix (Thermo Fisher Scientific). The sense and antisense primers of respective genes which specific for murine are mentioned in Table 1. Polymerase chain reaction (PCR) was performed by QuantStudio 3 Real-Time PCR System (Thermo Fisher Scientific) under these conditions: denaturation at 95 °C for 10 min, followed by 45 amplification cycles at 95 °C for 3 s, and annealing at the appropriate temperature for 30 s. The relative gene expression levels were calculated using the ΔΔCq method with normalization to β-actin as the reference gene [29].

### 2.8. Western Blotting Analysis

The RAW 264.7 cells were seeded in 6-well plate at the density of 2 × 10^6^ cells/well for 24 h. Cells were treated with the presence or absence of KM1608 at the concentration of 100 µg/mL. After multiple time-points, cells were collected and washed with Dulbecco's phosphate-buffered saline (DPBS), before lysed in radioimmunoprecipitation assay (RIPA) buffer supplemented with 1X protease inhibitor cocktail and 1 mM sodium orthovanadate (Na_3_VO_4_) phosphatase inhibitor to get the whole-cell extracts according to the manufacturer’s instructions. Protein concentration of each whole-cell extracts was determined using the Pierce™ BCA Protein Assay Kit (Thermo Scientific). The equal protein amounts of each whole-cell extracts (10 μg/lane) were separated by electrophoresis in a 10% sodium dodecyl sulfate-polyacrylamide gel and blotted onto PVDF transfer membranes. Epitope-specific primary antibodies included phospho-c-Jun NH2-terminal kinase (p-JNK), phospho-extracellular signal–regulated kinase (p-ERK), and β-actin conjugated with secondary antibodies (Cell Signaling, Boston, MA, USA) were used to label the target proteins. The bound antibodies were detected by PierceTM ECL Advance Western Blotting Detection Reagents (Thermo Scientific) and visualized with FUSION Solo Chemiluminescence System (PEQLAB Biotechnologie GmbH, Germany).

### 2.9. Statistical Analysis

Data are presented as the mean ± standard error of the mean (SEM) from three independent experiments. Statistical analyses were determined using SigmaStat software with one-way analysis of variance (ANOVA), followed by Tukey’s test. A *p*-value < 0.05 was considered statistically significant.

## 3. Results

### 3.1. Network Pharmacological Analyses of KM1608 Compounds

**Figure 1:**
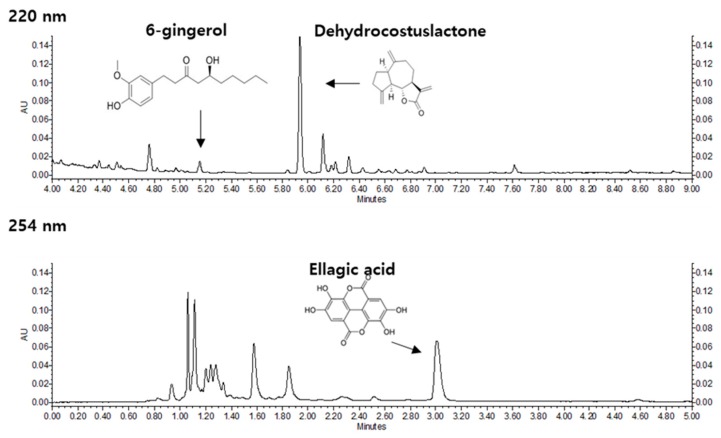
The HPLC profiles of KM1608. The peaks of 6-gingerol and dehydrocostus lactone at 220 nm, and ellagic acid at 254 nm in KM1608 were compared with those of their respective standard compounds.

The HPLC analysis showed a wide variety of phytochemicals in KM1608 (Figure 1). Among them, dehydrocostus lactone (DCL), ellagic acid (EA), and 6-gingerol (6G) in *S. lappa*, *T. chebula*, and *Z. officinale*, respectively, are active compounds possessing anti-inflammatory activity [30]. KM1608 contained 0.68–1.2%, 0.94–1.5%, and 0.1–0.2% of DCL, EA, and 6G, respectively.

**Figure 2:**
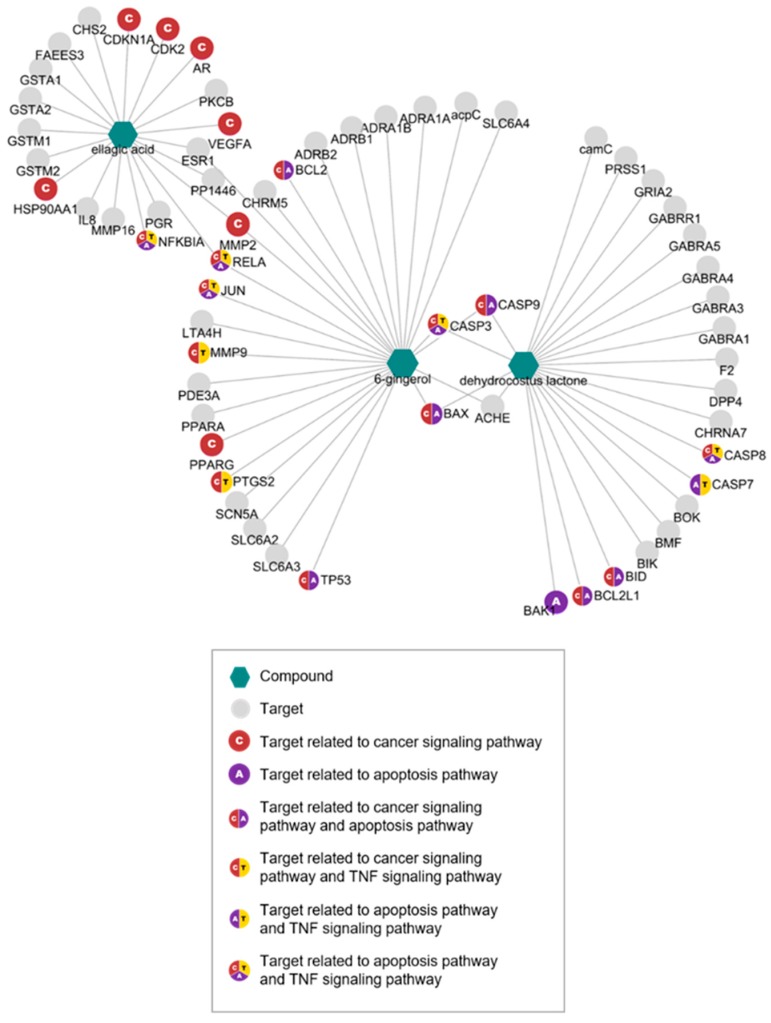
Compound-target gene network of active compounds of KM1608. Each node represents compound or target (explained in the box) and the edge represents the interaction between the compound and the target.

To determine potential targets and their mechanism of action in the immune system, we performed network pharmacological analyses. We identified 62 targets of the active compounds of KM1608 using an in silico model from TCMSP (Table 2). Overlapped targets between the compounds were investigated: ESR1, PP1446, MMP2, and RELA overlapped in EA and 6G; CASP3, CASP9, BAX, and ACHE overlapped in 6G and DCL; no overlapped targets were found between DCL and EA. KEGG analysis was conducted to determine potential pathways of the targets (Table 3). Among the targets, 22 genes were involved in cancer, apoptosis, and TNF signaling pathways, all of which are related to the immune response (Figure 2).

### 3.2. Stimulatory Effect of KM1608 on Nitric Oxide Production

**Figure 3:**
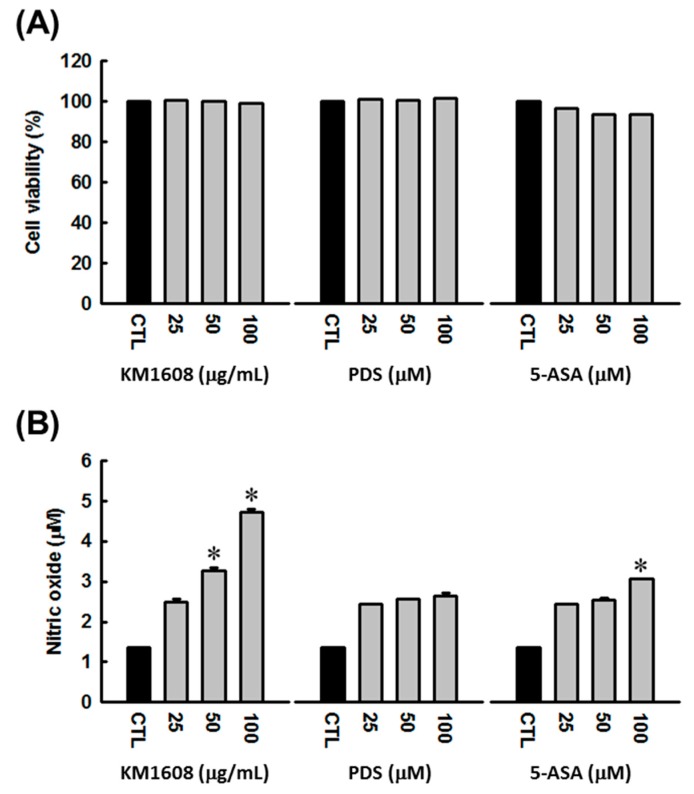
Stimulatory effect of KM1608 on nitric oxide production. (**A**) KM1608 cytotoxicity on RAW 264.7 cells. (**B**) Stimulatory effect of KM1608 on nitric oxide production in macrophages. RAW 264.7 cells were treated with phenol red-free medium containing 0.5% DMSO (control) or KM1608 at the indicated concentrations for 24 h. The cell culture supernatant was collected and mixed with Griess reagent at a ratio of 1:1 (*v/v*) and the absorbance was measured at 450 nm with a microplate reader. The concentration of nitrite, the stable product of nitric oxide metabolism, was calculated from a sodium nitrite standard reference curve. The experiments were conducted triplicate. ^∗^ *p* < 0.05 compared with the 0.5% DMSO treated group. KM1608: herbal formulation KM1608 extract, PDS: prednisolone, ASA: 5-aminosalicylic acid.

KM1608 was evaluated for its cytotoxicity on RAW 264.7 macrophages using the cell viability assay. As shown in Figure 3a, treatment with KM1608, up to the concentration of 100 µg/mL, did not suppress cell proliferation. The stimulatory effect of KM1608 on nitric oxide production was investigated by measuring the concentration of accumulated nitrite, the stable product of nitric oxide metabolism, released in cell culture medium. After KM1608 treatment at concentrations of 50 and 100 µg/mL for 24 h, the concentration of accumulated nitrite was increased in a dose-dependent manner (Figure 3b). Prednisolone (PDS) and 5-aminosalicylic acid (5-ASA) were used as reference drugs.

### 3.3. Effect of KM1608 on Cytokine Expression

**Figure 4:**
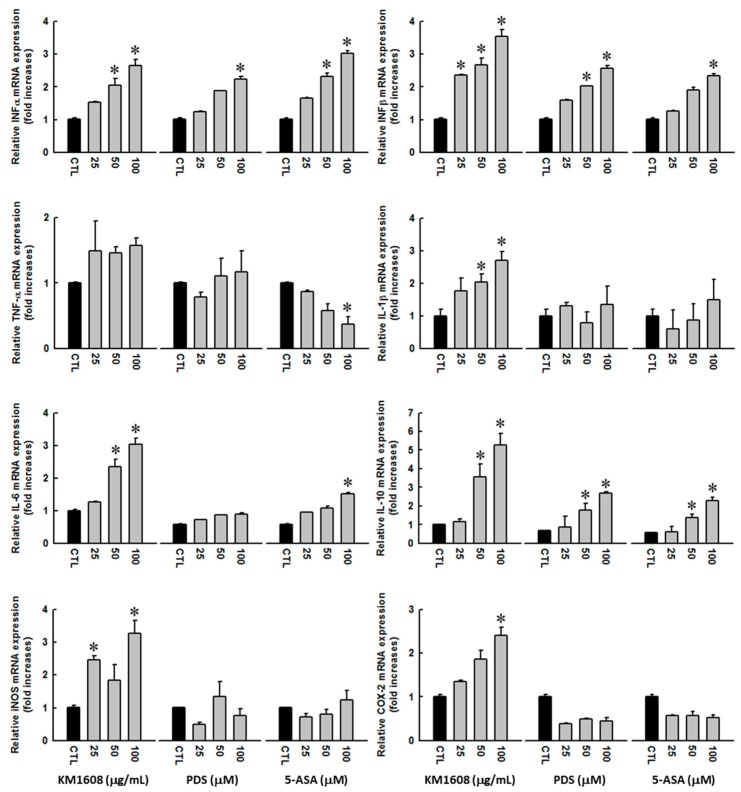
Effect of KM1608 on the expression of cytokines. RAW 264.7 cells were harvested 12 h post treatment with 0.5% DMSO (control) or KM1608 at the indicated concentrations. Total cellular RNA was isolated with TRIzol reagent and then reverse transcribed to cDNA. The reaction mixture was prepared by mixing cDNA with sense and antisense primers. PCR was performed using 45 amplification cycles. The relative gene expression levels were calculated by the ΔΔCq method. ^∗^ *p* < 0.05 compared with the 0.5% DMSO treated group. KM1608: herbal formulation KM1608 extract, PDS: prednisolone, ASA: 5-aminosalicylic acid.

**Figure A1:**
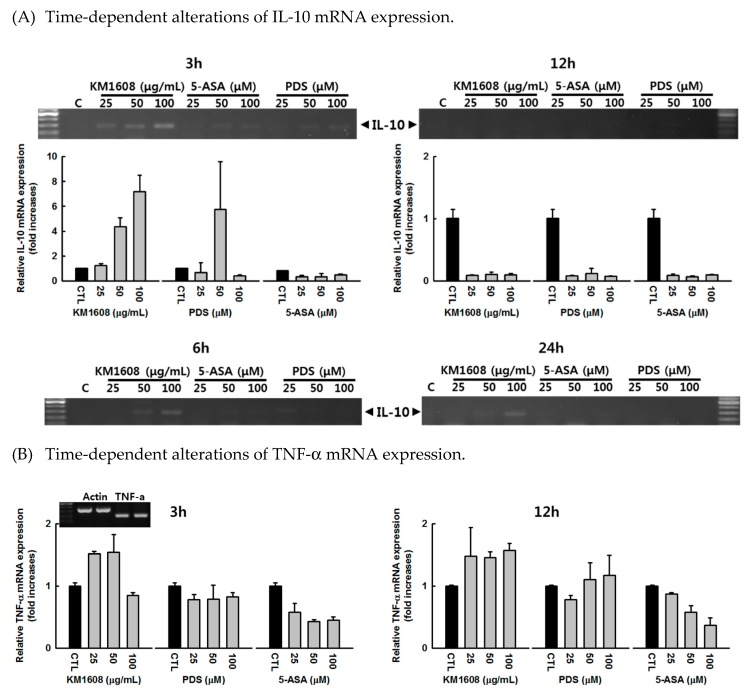

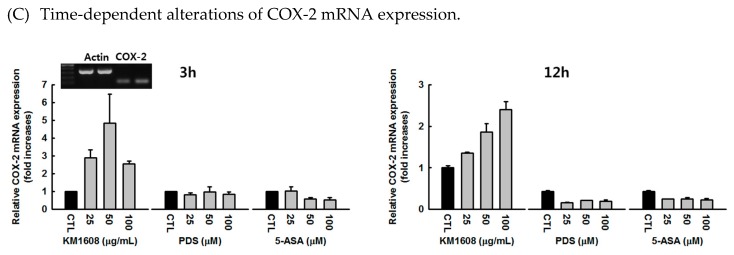
Time-dependent alterations of cytokine expression: (**A**) IL-10, (**B**) TNF-α, and (**C**) COX-2. RAW 264.7 cells were harvested at multiple time points after treatment with 0.5% DMSO (control) or KM1608 at the indicated concentrations. Total cellular RNA was isolated with TRIzol reagent and then reverse transcribed to cDNA. The reaction mixture was prepared by mixing cDNA with sense and antisense primers. PCR was performed using 45 amplification cycles. The relative gene expression levels were calculated by the ΔΔCq method. ^∗^ *p* < 0.05 compared with the 0.5% DMSO treated group. KM1608: herbal formulation KM1608 extract, PDS: prednisolone, ASA: 5-aminosalicylic acid.

An immune response is triggered and regulated by the participation of multiple cytokines at each stage. In this study, we performed qRT-PCR to evaluate the efficacy of KM1608 in stimulating the expression of selected cytokines in macrophage cells. After KM1608 treatment with the concentrations of 25–100 µg/mL for 12 h, the mRNA expression of interferons, interleukins, and pro-inflammatory cytokines in RAW 264.7 cells was significantly increased in a dose-dependent manner (Figure 4). The experimental conditions for gene changes were determined by analyzing various time-dependent alterations of mRNA expression through preliminary studies (Appendix A Figure A1(A)–(C)).

### 3.4. Effect of KM1608 on MAPKs Signaling Pathway

**Figure 5:**
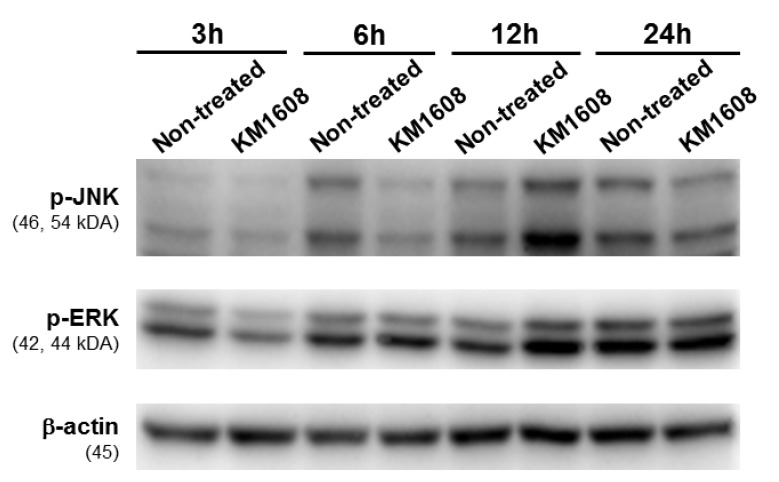
Western blot analysis for the time-dependent expression of mitogen-activated protein kinases (MAPKs) in RAW 264.7 macrophages after treated with KM1608.

We evaluated further the protein expression of mitogen-activated protein kinases (MAPKs), which involve in the regulation cytokine production. As a result, 12 h after treatment with KM1608 at the concentration of 100 µg/mL, the protein expression of p-JNK and p-ERK was increased comparing with non-treated group (Figure 5).

## 4. Discussion

The usage of natural products to support the treatment of chronic diseases is becoming more popular, leading to an increase of studies about the pharmacological effects of medicinal plants as well as their natural compounds for screening the new potential candidates. In this present study, we built up the herbal formulation named KM1608 with the ingredient include the extracts from three medicinal plants *S. lappa*, *T. chebula*, and *Z. officinale*. These herbs have been used for a long time in Asian folk medicine and also separately studied for bioactivities of each one. Thus, we questioned the combination pharmacological effects of the herbal extracts in KM1608.

In order to orientate the in vitro assays, network pharmacological analyses of KM1608 compounds were performed. From the HPLC analysis results, we determined three main compounds in the ingredient of KM1608 were dehydrocostus lactone (DCL), ellagic acid (EA), and 6-gingerol (6G). Results from network pharmacological analysis showed that these compounds target in several signaling pathways such as cancer, apoptosis, hepatitis B, neuroactive ligand-receptor interaction, ALS, nicotine addiction, viral carcinogenesis, p53, and TNF. In comparison with previous studies, dehydrocostus lactone from *S. lappa* presented the anticancer effects on human leukemia HL-60 cells [4] and human cervical carcinoma HeLa cells [9]. Ellagic acid form *T. chebula* was reported to suppress the proliferation of human osteosarcoma HOS-1 malignant cells [31]. The cytotoxic effects of 6-gingerol from *Z. officinale* were also mentioned on cancer cell lines included human hematopoetic leukemia HL-60 cells, murine solid sarcoma S180 cells, and peripheral blood mononuclear PBMC cells [32].

By using an in silico model from TCMSP and KEGG analysis, we identified that these compounds had the potential to take part in the interaction with the immune response signaling pathways. Later experimental results also showed that KM1608 stimulated the nitric oxide production on RAW 264.7 macrophages. Nitric oxide is synthesized during immune and inflammatory responses of the host against infectious agents. Nitric oxide is used as a toxin for the self-defense of the host or acts as an immunoregulatory mediator that participates in both innate and adaptive immune responses [33,34]. Our results also showed that the synthesis of NO was slightly stimulated by treatment with prednisolone (PDS) and 5-aminosalicylic acid (5-ASA), even though they were used as anti-inflammatory drugs. In fact, some previous researches mentioned that NO releasing is capable to enhance the therapeutic effect of anti-inflammatory drugs. The NCX 1022, a derivative of hydrocortisone, prevented the recruitment of granulocytes to the site of inflammation in C57BL6 mice by releasing NO [35]. In another study, treatment with MyoNovin and isosorbide dinitrate, two nitric oxide donor drugs, improve the anti-inflammatory effect of prednisone on dystrophic *mdx* mice [36]. Prednisolone was also mentioned that suppressed the proliferation of SaOS2, an osteosarcoma cell line, by stimulating the release of NO through the upregulation of iNOS [37].

The KM1608 stimulated the expression of both IFN-α and -β, and exhibited a strong effect on IFN-β. IFN-α and -β belong to type-I interferons (IFN), which induce the innate immune response as well as activate the adaptive immunity against virus infection. Type-I IFNs participate in the immune response by preventing virus replication as well as inducing the antiviral activities of natural killer cells, dendritic cells, and monocytes. They also promote the IL-10 expression in macrophages. Type-I IFNs not only trigger the antiviral immune response, but also downregulate this response to limit the tissue damage [38]. In addition, type-I IFNs participate in the antitumor activity by promoting the adaptive immune response of CD8+ T-cells which controls tumor growth. When delivered to tumor sites via monocytes, IFN-α suppresses the tumor growth and metastasis. IFN-β conjugates with tumor-targeting monoclonal antibodies also induces the antitumor immune response through the activation of dendritic cells [3].

In addition, the mRNA expression of three interleukins including IL-1β, IL-6, and IL-10 was also enhanced upon KM1608 treatment. However, KM1608 did not induce the expression of tumor necrosis factor-α (TNF-α). Different from other cytokines, IL-1β is synthesized from a precursor and requires activation by inflammasome [39]. IL-1β controls the recruitment of neutrophils to the infection site to destroy the damaged tissue [40]. IL-6 plays multiple roles in the immune response, such as recruitment of mononuclear cells, stimulation of B-cells as well as endothelial cells, and inhibition of T-cell apoptosis [41]. In contrast to IL-1β and IL-6, IL-10 acts as an anti-inflammatory cytokine that regulates the immune response to minimize host damage. IL-10 inhibits the activation of T-cells, monocytes, and macrophages to limit and terminate the inflammation [42]. These results seem to be contradictory when KM1608 enhanced the expressions of both pro- and anti-inflammatory cytokines at the same time. However, each cytokine play its own role in the immune response, whether it stimulates the inflammatory process or not. Our results showed that KM1608 enhanced the mRNA expressions of IFN-α, -β, and IL-10, consistent with the previous studies that type-I IFNs stimulate the IL-10 expression in macrophages [38].

In addition, we assessed the mRNA expression of iNOS and COX-2 in RAW 264.7 cells upon KM1608 treatment. KM1608 slightly stimulated the expression of these pro-inflammatory modulators. The role of iNOS in the synthesis of nitric oxide is well known; iNOS catalyzes the conversion of L-arginine to L-citrulline and NO [43]. In this study, we also found that KM1608 induces NO production by macrophages. NO is an important for host defense against infectious organisms. Moreover, NO can induce the cell function as well as regulate the growth and death of many immune cells, such as macrophages, neutrophils, T-cells, and natural killer cells [33]. In contrast to iNOS, COX-2 participates in the immune response by stimulating the cyclooxygenase pathway that leads to the synthesis of PGE2 [44]. PGE2 elicits vasodilatation and increases blood flow that facilitates the recruitment of neutrophils and macrophages to the infection site. PGE2 also promotes the production of a few pro-inflammatory cytokines, such as IL-17 and IL-13 [45].

In the previously published paper of Shin et al. [30], the results showed that the anti-inflammatory activity of KM1608 caused by the significant inhibition of nitric oxide synthesis as well as suppressing the expressions of inflammatory mediators such as IL-6, MCP-1, and TNF-α in lipopolysaccharides (LPS)-treated RAW264.7 cells. With the same material, the extract of herbal formulation KM1608, our recent studies suggested that KM1608 itself stimulated the expression of immune cytokines liked IFN-α, -β, IL-1β, -6, IL-10, iNOS, and COX-2 in RAW264.7 macrophages. We evaluated further the protein expression of MAPKs, which involve in the regulation cytokine production [46]. KM1608 stimulated the time-dependent protein expressions of phospho-c-Jun NH2-terminal kinase (p-JNK) and phospho-extracellular signal–regulated kinase (p-ERK). This result suggested that KM1608 might contribute to the up-regulation of cytokine synthesis through the MAPKs signaling pathway.

## 5. Conclusions

Network pharmacological analysis results showed that major compounds of KM1608 exhibit anticancer potential via immune signaling pathways. Both innate and adaptive immune responses are controlled by multiple signaling pathways using numerous complement proteins, cytokines, inflammatory mediators, and antibodies. Thus, we chose the most popular cytokines to study the stimulatory effect of KM1608 extract on the immune response. KM1608 induced the mRNA expression of IFN-α, IFN-β, IL-1β, IL-6, IL-10, iNOS, and COX-2 in RAW 264.7 macrophages after treatment at 25–100 μg/mL for 12 h. This effect might be caused by the stimulation of MAPKs signaling pathway through the phosphorylation of JNK and ERK. KM1608 also stimulated the synthesis of nitric oxide, but did not cause cytotoxicity in RAW 264.7 at the same concentration. As a result, KM1608 can be considered a potential candidate for preventive medicine to increase body resistance and support cancer treatment.

## Figures and Tables

**Table 1:** List of sense and antisense primers for quantitative real-time reverse transcription polymerase chain reaction (qRT-PCR).

Genes	Sense (5′→3′)	Antisense (5′→3′)
*IFN-* *α*	CCTGTGTGATGCAGGAACC	TCACCTCCCAGGCACAGA
*IFN-* *β*	ACTAGAGGAAAAGCAAGAGGA	CTGGTAAGTCTTCGAATGATG
*TNF-* *α*	ATAGCTCCCAGAAAAGCAAGC	CACCCCGAAGTTCAGTAGACA
*iNOS*	CATGCTACTGGAGGTGGGTG	CATTGATCTCCGTGACAGCC
*COX-2*	TCTGGAACATTGTGAACAACATC	AAGCTCCTTATTTCCCTTCACAC
*IL-1* *β*	ACCTGCTGGTGTGTGACGTT	TCGTTGCTTGGTTCTCCTTG
*IL-6*	TGGAGTCACAGAAGGAGTGGCTAAG	TCTGACCACAGTGAGGAATGTCCAC
*IL-10*	GTGAAGACTTTCTTTCAAACAAAG	CTGCTCCACTGCCTTGCTCTTATT
*β-actin*	TCACCCACACTGTGCCCATCTACGA	GGATGCCACAGGATTCCATACCCA

(IFN: interferon, TNF-α: tumor necrosis factor-α, iNOS: inducible nitric oxide synthase, and COX-2: cyclooxygenase-2, IL: interleukin,).

**Table 2:** Predicted or verified target genes of the active compounds of KM1608.

Targets of 6-gingerol (6G)	
Gene Name	Protein Name
*ACHE*	Acetylcholinesterase
*acpC*	Beta-lactamase
*ADRA1A*	Alpha-1A adrenergic receptor
*ADRA1B*	Alpha-1B adrenergic receptor
*ADRB1*	Beta-1 adrenergic receptor
*ADRB2*	Beta-2 adrenergic receptor
*BAX*	Apoptosis regulator BAX
*BCL2*	Apoptosis regulator Bcl-2
*CASP3*	Caspase-3
*CASP9*	Caspase-9
*CHRM5*	Muscarinic acetylcholine receptor M5
*ESR1*	Estrogen receptor
*JUN*	Transcription factor AP-1
*LTA4H*	Leukotriene A-4 hydrolase
*MMP2*	72-kDa type IV collagenase
*MMP9*	Matrix metalloproteinase-9
*PDE3A*	CGMP-inhibited 3’,5’-cyclic phosphodiesterase A
*PPARA*	Baculoviral IAP repeat-containing protein 5
*PPARG*	Peroxisome proliferator-activated receptor gamma
*PTGS2*	Prostaglandin G/H synthase 2
*RELA*	Transcription factor p65
*SCN5A*	Sodium channel protein type 5 subunit alpha
*SLC6A2*	Sodium-dependent noradrenaline transporter
*SLC6A3*	Sodium-dependent dopamine transporter
*SLC6A4*	Sodium-dependent serotonin transporter
*TP53*	Cellular tumor antigen p53
**Targets of Dehydrocostus Lactone (DCL)**	
**Gene Name**	**Protein Name**
*ACHE*	Acetylcholinesterase
*BAK1*	Bcl-2 homologous antagonist/killer
*BAX*	Apoptosis regulator BAX
*BCL2L1*	Bcl-2-like protein 1
*BID*	BH3-interacting domain death agonist
*BIK*	Bcl-2-interacting killer
*BMF*	Bcl-2-modifying factor
*BOK*	Bcl-2-related ovarian killer protein
*camC*	Cytochrome P450-cam
*CASP3*	Caspase-3
*CASP7*	Caspase-7
*CASP8*	Caspase-8
*CASP9*	Caspase-9
*CHRNA7*	Neuronal acetylcholine receptor protein, alpha-7 chain
*DPP4*	Dipeptidyl peptidase IV
*F2*	Thrombin
*GABRA1*	Gamma-aminobutyric acid receptor subunit alpha-1
*GABRA3*	Gamma-aminobutyric-acid receptor subunit alpha-3
*GABRA4*	Gamma-aminobutyric-acid receptor subunit alpha-4
*GABRA5*	Gamma-aminobutyric-acid receptor subunit alpha-5
*GABRR1*	Gamma-aminobutyric-acid receptor subunit rho-1
*GRIA2*	Glutamate receptor 2
*PRSS1*	Trypsin-1
**Targets of Ellagic Acid (EA)**	
**Gene Name**	**Protein Name**
*AR*	Androgen receptor
*CDK2*	Cell division protein kinase 2
*CDKN1A*	Cyclin-dependent kinase inhibitor 1
*CHS2*	Chitin synthase 2
*ESR1*	Estrogen receptor
*FAEES3*	Glutathione S-transferase P
*GSTA1*	Glutathione S-transferase A1
*GSTA2*	Glutathione S-transferase A2
*GSTM1*	Glutathione S-transferase Mu 1
*GSTM2*	Glutathione S-transferase Mu 2
*HSP90AA1*	Heat shock protein (HSP) 90
*IL8*	Interleukin-8
*MMP16*	Matrix metalloproteinase-16
*MMP2*	72-kDa type IV collagenase
*NFKBIA*	Nuclear factor kappa B inhibitor alpha
*PGR*	Progesterone receptor
*PKCB*	Protein kinase C beta type
*PP1446*	Insulin-like growth factor II
*RELA*	Transcription factor p65
*VEGFA*	Vascular endothelial growth factor A

**Table 3:** Result of Kyoto Encyclopedia Genes and Genomes (KEGG) pathway analysis on active compounds of KM1608 (adjusted *p*-value ≤ 0.05, combined score ≥ 30). ^†^ indicates the pathway relevant to immune responses.

KEGG Pathway (Homo sapiens)	Adjusted *p*-value	Combine d Score	Related Genes (Targets)
Cancer ^†^	3.22 × 10^−17^	90.44868	JUN; CDKN1A; HSP90AA1; MMP2; PTGS2; MMP9; RELA; VEGFA; NFKBIA; CASP9; AR; CASP8; CASP3; CDK2; BCL2; BAX; PPARG; BID; TP53; BCL2L1
Apoptosis ^†^	2.28 × 10^−14^	68.93754	JUN; RELA; NFKBIA; CASP9; CASP7; CASP8; CASP3; BCL2; BAX; BAK1; BID; TP53; BCL2L1
Hepatitis B	1.02 × 10^−12^	59.21799	NFKBIA; CASP9; JUN; CDKN1A; CASP8; CASP3; CDK2; BCL2; BAX; TP53; MMP9; RELA
Neuroactive ligand-receptor interaction	3.7 × 10^−12^	54.32502	GABRA1; GRIA2; PRSS1; GABRA5; GABRA4; CHRNA7; GABRA3; CHRM5; ADRB1; ADRB2; F2; ADRA1B; ADRA1A; GABRR1
Prostate cancer	1.69 × 10^−10^	46.99271	NFKBIA; CASP9; AR; HSP90AA1; CDKN1A; CDK2; BCL2; TP53; RELA
Amyotrophic lateral sclerosis (ALS)	8.21 × 10^−11^	44.6843	CASP9; GRIA2; CASP3; BCL2; BAX; BID; TP53; BCL2L1
Nicotine addiction	6.39 × 10^−10^	37.8611	GABRA1; GRIA2; GABRR1; GABRA5; GABRA4; CHRNA7; GABRA3
Viral carcinogenesis	9.31 × 10^−9^	36.97465	NFKBIA; JUN; CDKN1A; CASP8; CASP3; CDK2; BAX; BAK1; TP53; RELA
p53 signaling pathway	6.45 × 10^−10^	36.67846	CASP9; CDKN1A; CASP8; CASP3; CDK2; BAX; BID; TP53
Small cell lung cancer	3.49 × 10^−9^	36.25028	NFKBIA; CASP9; CDK2; BCL2; PTGS2; TP53; RELA; BCL2L1
TNF signaling pathway ^†^	2.08 × 10^−8^	33.21595	NFKBIA; JUN; CASP7; CASP8; CASP3; PTGS2; MMP9; RELA
